# A mathematical model for strigolactone biosynthesis in plants

**DOI:** 10.3389/fpls.2022.979162

**Published:** 2022-09-02

**Authors:** Abel Lucido, Oriol Basallo, Albert Sorribas, Alberto Marin-Sanguino, Ester Vilaprinyo, Rui Alves

**Affiliations:** ^1^Systems Biology Group, Department Ciències Mèdiques Bàsiques, Faculty of Medicine, Universitat de Lleida, Lleida, Spain; ^2^Institut de Recerca Biomédica de Lleida (IRBLleida), Lleida, Spain

**Keywords:** strigolactones, arbuscular mycorrhizal fungi, mathematical modeling, computational biology, feedback regulation, biosynthetic pathway

## Abstract

Strigolactones mediate plant development, trigger symbiosis with arbuscular mycorrhizal fungi, are abundant in 80% of the plant kingdom and help plants gain resistance to environmental stressors. They also induce germination of parasitic plant seeds that are endemic to various continents, such as *Orobanche* in Europe or Asia and *Striga* in Africa. The genes involved in the early stages of strigolactones biosynthesis are known in several plants. The regulatory structure and the latter parts of the pathway, where flux branching occurs to produce alternative strigolactones, are less well-understood. Here we present a computational study that collects the available experimental evidence and proposes alternative biosynthetic pathways that are consistent with that evidence. Then, we test the alternative pathways through *in silico* simulation experiments and compare those experiments to experimental information. Our results predict the differences in dynamic behavior between alternative pathway designs. Independent of design, the analysis suggests that feedback regulation is unlikely to exist in strigolactone biosynthesis. In addition, our experiments suggest that engineering the pathway to modulate the production of strigolactones could be most easily achieved by increasing the flux of β-carotenes going into the biosynthetic pathway. Finally, we find that changing the ratio of alternative strigolactones produced by the pathway can be done by changing the activity of the enzymes after the flux branching points.

## Introduction

Strigolactones (SLs) are a group of plant hormones that have pleiotropic effects. They were initially discovered as a germination stimulant of the parasitic plant *Striga* (Cook et al., 1966). Later, it became apparent that they are also responsible for promoting symbiotic interactions between plant and soil microbes (Akiyama et al., 2005) and controlling plant and root growth and development (Gomez-Roldan et al., 2008; Umehara et al., 2008).

Strigolactones were identified in most cereal crops’ root exudates and play a crucial role in host-parasite interactions (Bouwmeester et al., 2003). SLs induce hyphal branching in arbuscular mycorrhizal (AM) fungi leading to the establishment of symbiosis. The fungi colonize the root cortex and supply the root with inorganic nutrients in exchange for carbohydrates derived from photosynthesis (Akiyama et al., 2005). Aside from nutrients, AM fungi also contribute to water uptake under drought stress, apparently by increasing the production of indol acetic acid (Marulanda et al., 2009).

Several SLs exist, and two of the most important ones are orobanchol (ORO) and strigol (STR). In addition to their roles in plant development, ORO mediates the germination of *Orobanche*, and STR mediates the germination of *Striga*. These are two of the most critical parasitic plants whose germination is mediated by the SLs present in root cereal exudates. While *Striga* parasites are endemic in African soils and affect 70% of the continent’s cereal crops, *Orobanche* parasites are problematic in the Middle East, Europe, and North America, having important effects on leguminous crops (Bouwmeester et al., 2003; Yacoubou et al., 2021). With limited agricultural resources in Sub-Saharan Africa, *Striga* infestation and draughts remain essential obstacles to overcome for cereal production in the continent (Scholes and Press, 2008).

**FIGURE 1:**
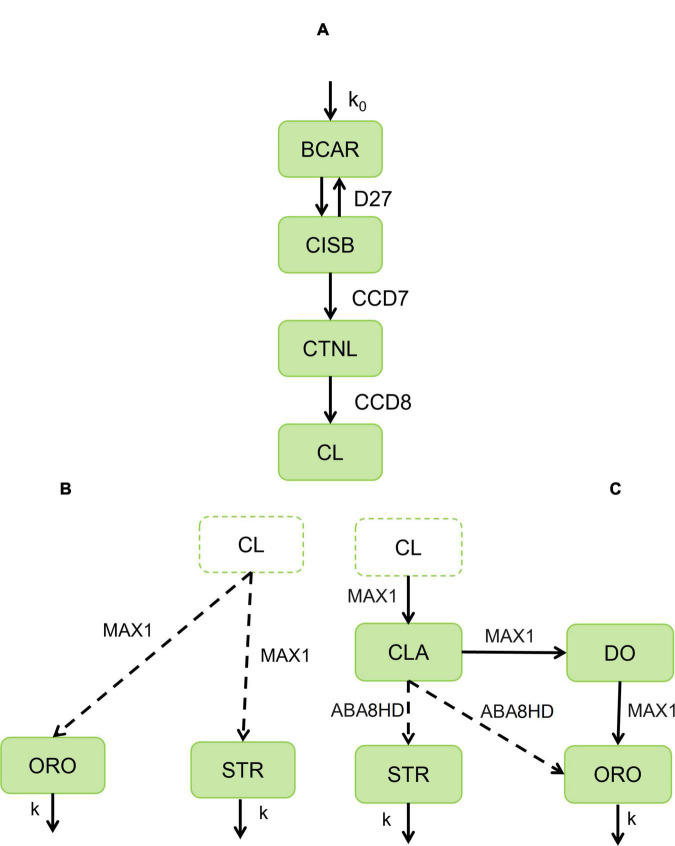
Alternative pathways for the biosynthesis of strigolactones in cereals. **(A)** Biosynthetic pathway of strigolactones from all-trans-β –carotene (BCAR) to be catalyzed by D27 to produce 9-*cis*-β –carotene (CISB). Then CCD7 will catalyze CISB to obtain 9-*cis*-β –apo-10′-carotenal (CTNL), which will be further catalyzed to carlactone (CL) by CCD8. **(B)** Then we assume that MAX1 will catalyze CL to produce orobanchol-type (ORO) and strigol-type (STR). **(C)** CL is further catalyzed by MAX1 to carlactonic acid (CLA). CLA is converted and distributed to 4-deoxyorobanchol (DO) by MAX1 and ORO and 5-deoxystrigol (5DS) by ABA. Then DO is further converted into orobanchol. The boxes represent metabolite concentration, and the arrows are enzyme reactions, where the substrate (beginning of the arrow) transforms into a product (end of the arrow). Solid arrows indicate experimentally confirmed enzymes involved in the catalysis. Dashed arrows indicate the maize enzyme with the highest homology to those catalyzing the reaction in other cereals. Model AB (AC) couples modules A and B **(C)**. Parameters: influx of BCAR (k_0_), proportion of orobanchol-type (ω), turnover number (k_*cati*_), Michaelis-Menten’s constant (K_*Mi*_) with respect to enzyme concentration i = D27, CCD7, CCD8, MAX1, ABA.

Due to the critical roles of STR and ORO in plant development and crop yields, it is crucial to elucidate the biosynthetic steps for the various SLs and understand their regulation. All-trans-β –carotene (BCAR) is the initial substrate for the SLs biosynthesis pathway. The initial catalytic steps that go from BCAR to carlactone (CL) are well-characterized in cereals (Figure 1). The individual biosynthetic steps from CL to produce STR or ORO are less clear. Zhang et al. (2014) reported that rice MAX1 homolog Os900 and Os1400 catalyze alternative steps that transform CL either into ent-2′-epi-5-deoxystrigol (STR) or ORO. In concurrent experiments, Yoneyama et al. (2018) only detect that Arabidopsis MAX1, Os900, and Os1400 convert CL to carlactonic acid (CLA), which is then converted into 4-deoxyorobanchol (DO), suggesting that MAX1 can catalyze at least three of the steps that transform CLA into either STR or ORO. Clarifying the most likely reaction steps in this section of the ORO and STR biosynthetic pathway enables targeted and more effective genome manipulation toward potentiating the production of either STR or ORO.

Mathematical modeling provides a set of tools that can be helpful to differentiate between alternative reaction structures of a pathway. When significant amounts of quantitative data are available, statistical methods and optimization can be used to find relationships between variable and assign probabilities to alternative reaction structures (Shah et al., 2009; Guillén-Gosálbez et al., 2013; Su et al., 2013; Wang et al., 2022). If, on the other hand, no quantitative information is available, Boolean networks provide a reasonable approach to distinguish between alternative pathway structures (Mehra et al., 2004; Dealy et al., 2005; Schwab et al., 2020).

In the middle ground where, as is our case, some information is available, differential equation models provide a good formalism to work with for structure comparison. In general, alternative models for the same pathway can be constructed (Alves and Savageau, 2000; Igoshin et al., 2008; Alves et al., 2021). Then, their dynamics can be characterized and compared to experimental observations to elucidate which pathway structure is consistent with experimental observations (Alves et al., 2004a,b; Alves and Sorribas, 2007). As such, models can be essential tools to predict undetermined phenomena, test hypotheses, and evaluate the potential consequences of alternative actions (Torres and Santos, 2015). This methodology can help differentiate the dynamic behavior of alternative mechanisms involved in the production of SLs in plants. In this study, we will apply this methodology to construct two alternative models for the biosynthesis of STR and ORO that are consistent with the experimental observations reported above. We then interrogate the models by performing *in silico* experiments to identify potential differences in their dynamic behavior of the alternative models. The differences between the two pathways in relation to producing ORO and STR can be used to propose additional experiments and leverage this information to further clarify the structure and regulation of SLs biosynthesis.

## Materials and methods

### Pathway reconstruction

The available experimental information is consistent with two alternative structures for the SLs biosynthesis pathway, as described in the Section “Introduction.” A conceptual representation of each alternative is illustrated in Figure 1.

The core pathway of strigolactone biosynthesis is consensual among several studies (Alder et al., 2012; Waters et al., 2012; Zhang et al., 2014; Yoneyama et al., 2018; Mashiguchi et al., 2021) and common to both alternative models (Figure 1A). This core of reactions describes the transformation of all-trans-β-carotene (BCAR) into carlactone (CL). The individual steps of the core are illustrated in Figure 1A. Healthy plants can provide a supply of BCAR to the strigolactone biosynthesis pathway. As such, we assume that the plant produces BCAR at a constant rate, k0. This constant rate can be modulated to account for the variability in BCAR availability observed in plants. BCAR is used by β –carotene isomerase Dwarf27 (D27) to make 9-*cis*-β-carotene (CISB) (Alder et al., 2012). Then, carotenoid cleavage dioxygenase 7 (CCD7) cleaves CISB into all-trans-β –apo-10′-carotenal (CTNL) and β –ionone. Subsequently, the enzyme CCD8 converts CTNL into CL.

We know less about the individual steps that convert CL into the subsequent intermediates of SL biosynthesis. The simplest scenario (Figure 1B) consistent with available experimental information is to consider that a single multi-step P450 enzyme uses CL and synthesizes STR and ORO (Zhang et al., 2014).

Here, we introduce a parameter 0 ≤ ω ≤ 1. In the absence of additional experimental information, this parameter determines which percentage of flux $f=\frac{V_{\max M\,A\,X\,1}\,\left[C\,L\right]}{K_{M\,M\,A\,X\,1}+\left[C\,L\right]}$ is drawn from the CL pool to produce ORO (ω × *f*) and which goes to STR [(1 − ω) × *f*]. The model also assumes that ORO and STR diffuse away from the production site at a rate that is proportional to their respective concentrations. The proportionality constants for this diffusion are defined by k.

Several studies suggest that the lower part of the biosynthetic SL pathway may be different (Figure 1C). It is known that CL is converted to carlactonic acid (CLA) by MAX1 in *Arabidopsis thaliana* (CYP711A1/AtMAX1) and *Oryza sativa* (CYP711A2/Os900 and CYP711A3/Os1400) (Yoneyama et al., 2018). Moreover, CLA is further converted to 4-deoxyorobanchol (DO) by CYP711A2. Then, DO is converted into orobanchol by CYP711A3 (Yoneyama et al., 2018). In parallel, CLA is converted to ORO in cowpea and tomato by CYP722 (Wakabayashi et al., 2019). Furthermore, CYP722S seems to convert CLA into a strigol in cotton (Wakabayashi et al., 2020). In maize, the closest sequence ortholog for CYP722C is abscisic acid 8′-hydroxylase (ABA8HD).

Because ABA8HD can potentially catalyze the alternative conversion of CLA into either ORO or STR, we introduce a parameter 0 ≤ ϕ ≤   1. This parameter determines which percentage of flux $f^{*}=\frac{V_{\max A\,B\,A}\,\left[C\,L\,A\right]}{K_{M\,A\,B\,A}+\left[C\,L\,A\right]}$ is drawn from the CLA pool to produce ORO (ϕ×f*) and which is used to produce STR [(1 − ϕ) × f*].

### Model building and assembly

The mathematical model was built based on the two alternative pathways presented in Figure 1. We coupled the core pathway (Figure 1A) to the use of CL by MAX1 to synthesize the formation of both strigol-type and orobanchol-type strigolactones (Figure 1B). This created the first model (Model AB hereafter). On the other hand, Model AC was created by coupling the core pathway with the pathway shown in Figure 1C, which is based on several experimental studies (Yoneyama et al., 2018; Wakabayashi et al., 2019, 2020). Here, CL was used to synthesize CLA. CLA is then transformed into ORO and STR.

All the reactions are described using a Michaelis-Menten approximation $\frac{k_{c\,a\,t\,i}\,\left[i\right]\,\left[S\right]}{K_{M\,i}+\left[S\right]}$. In this approximation, the flux of each process depends on the concentrations of substrate [*S*] and enzyme [*i*]. The parameters in the equation are the turnover number k_*cat*_*_*i*_*, and the Michaelis-Menten constant K_*Mi*_, where *i* is any of the enzymes in the pathway (that is *i* = [*D*27, *CCD*7, *CCD*8, *MAX*1, *ABA*8*HD*]). Note that V_*max*_*_*i*_*, the maximum reaction rate, is equivalent to the product of k_*cat*_*_*i*_* and [*i*]. Equation 1 shows the system of ordinary differential equations (ODEs) that describe the dynamics of the core pathway:


(1) $$\left[B\,\dot{C}\,A\,R\right]=$$

$$k_{0}-\frac{k_{c\,a\,t\,D\,27\,A}\,\left[D\,27\right]\,\left[B\,C\,A\,R\right]}{K_{M\,D\,27}+\left[B\,C\,A\,R\right]}+\frac{k_{c\,a\,t\,D\,27\,B}\,\left[D\,27\right]\,\left[C\,I\,S\,B\right]}{K_{M\,D\,27}+\left[C\,I\,S\,B\right]}$$

$$\left[C\,\dot{I}\,S\,B\right]=\frac{k_{c\,a\,t\,D\,27\,A}\,\left[D\,27\right]\,\left[B\,C\,A\,R\right]}{K_{M\,D\,27}+\left[B\,C\,A\,R\right]}$$

$$-\frac{k_{c\,a\,t\,D\,27\,B}\,\left[D\,27\right]\,\left[C\,I\,S\,B\right]}{K_{M\,D\,27}+\left[C\,I\,S\,B\right]}-\frac{k_{c\,a\,t\,C\,C\,D\,7}\,\left[C\,C\,D\,7\right]\,\left[C\,I\,S\,B\right]}{K_{M\,C\,C\,D\,7}+\left[C\,I\,S\,B\right]}$$

$$\left[C\,\dot{T}\,N\,L\right]=\frac{k_{c\,a\,t\,C\,C\,D\,7}\,\left[C\,C\,D\,7\right]\,\left[C\,I\,S\,B\right]}{K_{M\,C\,C\,D\,7}+\left[C\,I\,S\,B\right]}-\frac{k_{c\,a\,t\,C\,C\,D\,8}\,\left[C\,C\,D\,8\right]\,\left[C\,T\,N\,L\right]}{K_{M\,C\,C\,D\,8}+\left[C\,T\,N\,L\right]}$$

$$\left[\dot{C}\,L\right]=\frac{k_{c\,a\,t\,C\,C\,D\,8}\,\left[C\,C\,D\,8\right]\,\left[C\,T\,N\,L\right]}{K_{M\,C\,C\,D\,8}+\left[C\,T\,N\,L\right]}-\frac{V_{\max M\,A\,X\,1}\,\left[C\,L\right]}{K_{M\,M\,A\,X\,1}+\left[C\,L\right]}$$

Next, Equation 2 describes the dynamics of the series of processes shown in Figure 1B:


(2) $$\left[O\,\dot{R}\,O\right]=\omega \,\frac{V_{\max M\,A\,X\,1}\,\left[C\,L\right]}{K_{M\,M\,A\,X\,1}+\left[C\,L\right]}-k\,\left[O\,R\,O\right]$$

$$\left[S\,\dot{T}\,R\right]=\left(1-\omega \right)\,\frac{V_{\max M\,A\,X\,1}\,\left[C\,L\right]}{K_{M\,M\,A\,X\,1}+\left[C\,L\right]}-k\,\left[S\,T\,R\right]$$

Then, Equation 3 describes the dynamics of the series of processes described in Figure 1C:


(3) $$\left[C\,\dot{L}\,A\right]=\frac{V_{\max M\,A\,X\,1}\,\left[C\,L\right]}{K_{M\,M\,A\,X\,1}+\left[C\,L\right]}-\frac{V_{\max M\,A\,X\,1}\,\left[C\,L\,A\right]}{K_{M\,M\,A\,X\,1}+\left[C\,L\,A\right]}$$

$$-\frac{V_{\max A\,B\,A\,8\,H\,D}\,\left[C\,L\,A\right]}{K_{M\,A\,B\,A\,8\,H\,D}+\left[C\,L\,A\right]}$$

$$\left[\dot{D}\,O\right]=\frac{V_{\max M\,A\,X\,1}\,\left[C\,L\,A\right]}{K_{M\,M\,A\,X\,1}+\left[C\,L\,A\right]}-\frac{V_{\max M\,A\,X\,1}\,\left[D\,O\right]}{K_{M\,M\,A\,X\,1}+\left[D\,O\right]}$$

$$\left[O\,\dot{R}\,O\right]=\phi \,\frac{V_{\max A\,B\,A\,8\,H\,D}\,\left[C\,L\,A\right]}{K_{M\,A\,B\,A\,8\,H\,D}+\left[C\,L\,A\right]}+\frac{V_{\max M\,A\,X\,1}\,\left[D\,O\right]}{K_{M\,M\,A\,X\,1}+\left[D\,O\right]}-k\,\left[O\,R\,O\right]$$

$$\left[S\,\dot{T}\,R\right]=\left(1-\phi \right)\,\frac{V_{\max A\,B\,A\,8\,H\,D}\,\left[C\,L\,A\right]}{K_{M\,A\,B\,A\,8\,H\,D}+\left[C\,L\,A\right]}-k\,\left[S\,T\,R\right]$$

By combining Equations 1, 2, we obtain the ODEs that describe the dynamic behavior of Model AB. Combining Equations 1, 3, generates the ODEs that characterize the dynamic behavior of Model AC. Given that the pathway isn’t fully established, data for parameter estimation were obtained from different plants in several databases. We started with BRENDA (Chang et al., 2021) and complemented the information in that database by searching the primary literature. We provide the parameter values for the various processes in Table 1, together with the experimental references used to estimate those parameters. Parameter values for the models were obtained from the literature (Cutler et al., 2000; Waters et al., 2012; Harrison et al., 2015). Given that the quantitative information available for the pathway is limited, we made several approximations to estimate parameter values that are not available in the literature (Tables 1, 2). As a result, and while we had maize in focus while modeling, the models are expected to be a reasonable representation of SL biosynthesis in cereals.

**TABLE 1:** Parameter values for Model AB.

Notation	Original values	Unit	References and organism
k_0_	1 × 10^–8^*	mM s^–1^	
k_*cat D27A*_	68	s^–1^	(Harrison et al., 2015) Rice
D27	1.06 × 10^–7^*	mM	(Waters et al., 2012) Arabidopsis
K_*M D27*_	0.26*	mM	(Harrison et al., 2015) Rice
k_*cat D27B*_	34	s^–1^	(Harrison et al., 2015) Rice
k_*cat CCD7*_	26000	s^–1^	
CCD7	1.06 × 10^–7^	mM	(Waters et al., 2012) Arabidopsis
K_*M CCD8*_	0.0087	mM	(Harrison et al., 2015) Rice
k_*cat CCD8*_	0.18	s^–1^	(Harrison et al., 2015) Rice
CCD8	1.06 × 10^–6^*	mM	(Waters et al., 2012) Arabidopsis
K_*M CCD8*_	0.0092	mM	(Harrison et al., 2015) Rice
ω	0.5	–	
V_*max MAX1*_	6.08 × 10^–6^	mM/s	
K_*M MAX1*_	0.0005	mM	(Waters et al., 2012) Arabidopsis
k	1	s^–1^	

*The values for these parameters were adjusted to keep BCAR concentrations within experimentally determined values and maintain steady state stability (Supplementary Figure 1). Values found in the literature are: k_0_ = 3.7 ×10^–14^, D27 = 1.06 × 10^–8^ , K_*MD27*_ = 0.00026, CCD8 = 1.06 × 10^–7^.

**TABLE 2:** Parameter values for Model AC.

Notation	Original values	Unit	References
k_0_	1 × 10^–8^*	mM	
k_*cat D27A*_	68	s^–1^	(Harrison et al., 2015) Rice
D27	1.06 × 10^–7^*	mM	(Waters et al., 2012) Arabidopsis
K_*M D27*_	0.26*	mM	(Harrison et al., 2015) Rice
k_*cat D27B*_	34	s^–1^	(Harrison et al., 2015) Rice
k_*cat CCD7*_	26000	s^–1^	
CCD7	1.06 × 10^–7^	mM	(Waters et al., 2012) Arabidopsis
K_*M CCD7*_	0.0087	mM	(Harrison et al., 2015) Rice
k_*cat CCD8*_	0.18	s^–1^	(Harrison et al., 2015) Rice
CCD8	1.06 × 10^–6^*	mM	(Waters et al., 2012) Arabidopsis
K_*M CCD8*_	0.0092	mM	(Harrison et al., 2015) Rice
V_*max MAX1*_	6.08 × 10^–6^	mM/s	
K_*M MAX1*_	0.0005	mM	(Waters et al., 2012) Arabidopsis
V_*max ABA8HD*_	3.8 × 10^–9^	mM/s	(Cutler et al., 2000) Arabidopsis
K_*M ABA8HD*_	0.016	mM	(Cutler et al., 2000) Arabidopsis
k	1	s^–1^	

*The values for these parameters were adjusted to keep BCAR concentrations within experimentally determined values and maintain steady state stability (Supplementary Figure 2). Values found in the literature are: k_0_ = 3.7 ×10^–14^, D27 = 1.06 × 10^–8^ , K_*MD27*_ = 0.00026, CCD8 = 1.06 × 10^–7^.

### Including feedback regulation in the model

Negative feedback by pathway intermediates or products regulates the flux going through most biosynthetic pathways. The most common form of feedback regulation is overall feedback, also known as end-product inhibition, where the final product of the pathway inhibits the reaction of the first enzyme. In addition, when a biosynthetic pathway is branched, it is also typical that the metabolite at the branching point inhibits the first reaction of the pathway and that the final product of each branch inhibits the reaction of the branch and, sometimes, activates the first reaction of the opposite branch (Alves and Savageau, 2000). To the best of our knowledge, no such feedback is known to occur in strigolactone biosynthesis. Because this could be a consequence of the pathway being poorly characterized, we wanted to test the effect of potential feedback on the dynamics of the alternative models.

To describe potential feedback regulation in the system and in the absence of specific mechanistic information, we use the power-law formalism. This formalism allows us to mathematically approximate the dynamic effect of a variable on a process (Alves et al., 2008). We consider overall feedback inhibition, in which the end-product of the pathway inhibits the initial reaction in the pathway (Alves and Savageau, 2000). In our case, that is the reaction driven by D27, producing CISB from BCAR. The possibility of feedback regulated by the metabolite in the branching point (CL) is also included. Thus, we have the feedback regulated by CL, ORO, and STR added to Equation 1 by multiplying [CL]^*fcll*^, [ORO]^*foro*1^, and [STR]^*fstr*1^ into the expression $\frac{k_{c\,a\,t\,D\,27\,A}\,\left[D\,27\right]\,\left[B\,C\,A\,R\right]}{K_{M\,D\,27}+\left[B\,C\,A\,R\right]}$.

We also considered the possibility of other potential feedback interactions. First, we allowed ORO and STR to inhibit the reaction catalyzed by MAX1 (this enzyme uses CL to produce ORO and STR). This was implemented by multiplying either [ORO]^foro2^ or [STR]^fstr2^ to the expressions that describe the direct production of ORO or STR, respectively. The feedback parameters are allowed to range from –1 to 0 (for negative feedback; 0 to 1 if it were positive feedback). This excludes cooperativity. Sometimes, kinetic orders beyond one are necessary for some qualitative behaviors to appear.

This leads to the ODEs described in Equations 4, 5:


(4) $$\left[B\,\dot{C}\,A\,R\right]=$$

$$k_{0}-\frac{k_{c\,a\,t\,D\,27\,A}\,\left[D\,27\right]\,\left[B\,C\,A\,R\right]}{K_{M\,D\,27}+\left[B\,C\,A\,R\right]}\,{\left[C\,L\right]}^{f_{c\,l}}\,{\left[O\,R\,O\right]}^{f_{o\,r\,o\,1}}\,{\left[S\,T\,R\right]}^{f_{s\,t\,r\,1}}$$

$$+\frac{k_{c\,a\,t\,D\,27\,B}\,\left[D\,27\right]\,\left[C\,I\,S\,B\right]}{K_{M\,D\,27}+\left[C\,I\,S\,B\right]}$$

$$\left[C\,\dot{I}\,S\,B\right]=\frac{k_{c\,a\,t\,D\,27\,A}\,\left[D\,27\right]\,\left[B\,C\,A\,R\right]}{K_{M\,D\,27}+\left[B\,C\,A\,R\right]}\,{\left[C\,L\right]}^{f_{c\,l}}\,{\left[O\,R\,O\right]}^{f_{o\,r\,o\,1}}\,{\left[S\,T\,R\right]}^{f_{s\,t\,r\,1}}$$

$$-\frac{k_{c\,a\,t\,D\,27\,B}\,\left[D\,27\right]\,\left[C\,I\,S\,B\right]}{K_{M\,D\,27}+\left[C\,I\,S\,B\right]}-\frac{k_{c\,a\,t\,C\,C\,D\,7}\,\left[C\,C\,D\,7\right]\,\left[C\,I\,S\,B\right]}{K_{M\,C\,C\,D\,7}+\left[C\,I\,S\,B\right]}$$

$$\left[C\,\dot{T}\,N\,L\right]=$$

$$\frac{k_{c\,a\,t\,C\,C\,D\,7}\,\left[C\,C\,D\,7\right]\,\left[C\,I\,S\,B\right]}{K_{M\,C\,C\,D\,7}+\left[C\,I\,S\,B\right]}-\frac{k_{c\,a\,t\,C\,C\,D\,8}\,\left[C\,C\,D\,8\right]\,\left[C\,T\,N\,L\right]}{K_{M\,C\,C\,D\,8}+\left[C\,T\,N\,L\right]}$$

$$\left[\dot{C}\,L\right]=\frac{k_{c\,a\,t\,C\,C\,D\,8}\,\left[C\,C\,D\,8\right]\,\left[C\,T\,N\,L\right]}{K_{M\,C\,C\,D\,8}+\left[C\,T\,N\,L\right]}$$

$$-\omega \,\frac{V_{\max M\,A\,X\,1}\,\left[C\,L\right]}{K_{M\,M\,A\,X\,1}+\left[C\,L\right]}\,{\left[O\,R\,O\right]}^{f_{o\,r\,o\,2}}$$

$$-\left(1-\omega \right)\,\frac{V_{\max M\,A\,X\,1}\,\left[C\,L\right]}{K_{M\,M\,A\,X\,1}+\left[C\,L\right]}\,{\left[S\,T\,R\right]}^{f_{s\,t\,r\,2}}$$

(5) $$\left[O\,\dot{R}\,O\right]=\omega \,\frac{V_{\max M\,A\,X\,1}\,\left[C\,L\right]}{K_{M\,M\,A\,X\,1}+\left[C\,L\right]}\,{\left[O\,R\,O\right]}^{f_{o\,r\,o\,2}}-k\,\left[O\,R\,O\right]$$

$$\left[S\,\dot{T}\,R\right]=\left(1-\omega \right)\,\frac{V_{\max M\,A\,X\,1}\,\left[C\,L\right]}{K_{M\,M\,A\,X\,1}+\left[C\,L\right]}\,{\left[S\,T\,R\right]}^{f_{s\,t\,r\,2}}-k\,\left[S\,T\,R\right]$$

Furthermore, we also considered the possibility that ORO and STR could regulate the fluxes directly after the branching points from CLA in model AC. In the same model, we also consider the possibility that CLA could negatively regulate the initial reaction of the pathway catalyzed by D27. The dynamics of these feedbacks are described by Equation 6:


(6) $$\left[C\,\dot{L}\,A\right]=\frac{V_{\max M\,A\,X\,1}\,\left[C\,L\right]}{K_{M\,M\,A\,X\,1}+\left[C\,L\right]}-\frac{V_{\max M\,A\,X\,1}\,\left[C\,L\,A\right]}{K_{M\,M\,A\,X\,1}+\left[C\,L\,A\right]}\,{\left[O\,R\,O\right]}^{f_{o\,r\,o\,4}}$$

$$-\phi \,\frac{V_{\max A\,B\,A}\,\left[C\,L\,A\right]}{K_{M\,A\,B\,A}+\left[C\,L\,A\right]}\,{\left[O\,R\,O\right]}^{f_{o\,r\,o\,3}}$$

$$-\left(1-\phi \right)\,\frac{V_{\max A\,B\,A}\,\left[C\,L\,A\right]}{K_{M\,A\,B\,A}+\left[C\,L\,A\right]}\,{\left[S\,T\,R\right]}^{f_{s\,t\,r\,3}}$$

$$\left[\dot{D}\,O\right]=\frac{V_{\max M\,A\,X\,1}\,\left[C\,L\,A\right]}{K_{M\,M\,A\,X\,1}+\left[C\,L\,A\right]}\,{\left[O\,R\,O\right]}^{f_{o\,r\,o\,4}}$$

$$-\frac{V_{\max M\,A\,X\,1}\,\left[D\,O\right]}{K_{M\,M\,A\,X\,1}+\left[D\,O\right]}$$

$$\left[O\,\dot{R}\,O\right]=\phi \,\frac{V_{\max A\,B\,A}\,\left[C\,L\,A\right]}{K_{M\,A\,B\,A}+\left[C\,L\,A\right]}\,{\left[O\,R\,O\right]}^{f_{o\,r\,o\,3}}$$

$$+\frac{V_{\max M\,A\,X\,1}\,\left[D\,O\right]}{K_{M\,M\,A\,X\,1}+\left[D\,O\right]}-k\,\left[O\,R\,O\right]$$

$$\left[S\,\dot{T}\,R\right]=\left(1-\phi \right)\,\frac{V_{\max A\,B\,A\,8\,H\,D}\,\left[C\,L\,A\right]}{K_{M\,A\,B\,A\,8\,H\,D}+\left[C\,L\,A\right]}\,{\left[S\,\dot{T}\,R\right]}^{f_{s\,t\,r\,3}}-k\,\left[S\,T\,R\right]$$

### Quality control of models

**FIGURE 2:**
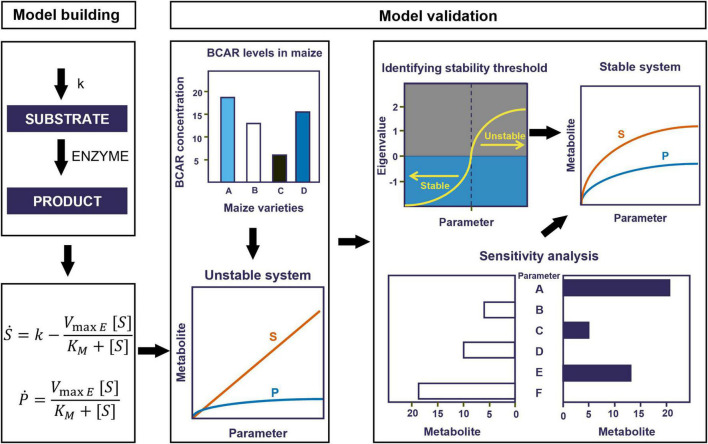
Workflow for model building and validation. First, we translate the conceptual models described in Figure 1 into systems of ordinary differential equations (ODEs). Second, we validate the models by comparing their behavior to known experimental results. Where inconsistent, we adjusted the influx of BCAR (which is represented by k_0_) to reach the appropriate experimental levels. If this leads to unstable steady states, we further optimize parameters to maintain the substrate close to experimentally compatible levels and stabilize the steady state.

We evaluated the quality of the resulting models. In brief, biological systems are robust to changes in parameter values, and models that are representative of physiological situations should have low sensitivities to most parameters (Savageau, 1975). In addition, the steady-state generated by these models needs to be stable (Savageau, 1976). Thus, the fundamental quality indicators of models are that they produce stable and robust steady states. We tested all our models using these indicators as described below. Figure 2 summarizes the process of model building and improvement.

#### Stability analysis

Steady state stability can be determined by analyzing the eigenvalue of the Jacobian matrix. First, we get the partial derivative of each equation with respect to dependent variables to get the Jacobian matrix. Next, we solve for the eigenvalue of the Jacobian matrix in which it is stable for negative eigenvalue and unstable for positive eigenvalue.

#### Sensitivity analysis

By definition, sensitivity analysis measures how much a variable changes if one of the parameters in the system is varied by a certain amount (Voit, 2013). The robustness of a steady state can be determined through sensitivity analysis. The more sensitive a variable is to a parameter change, the less robust the system is.

Sensitivity analysis identifies regions in the model where minor inaccuracies in parameter values can lead to almost unpredictable results. Consequently, this analysis also allowed us to identify components of the model that may be problematic due to unusually high sensitivity values (Savageau, 1975; Voit, 2013). In addition, this analysis allowed us to understand how the variables of the system depended on its parameters, therefore providing information about potentially helpful and relevant regulatory targets.

Mathematically, the sensitivity of a variable to a parameter can be calculated from


(7) $$\bar{S}\,\left(X,p\right)=\frac{\partial X/X}{\partial p/p}=\frac{\partial \log X}{\partial \log p}.$$

We performed a sensitivity analysis on all our models, as described in Comas et al. (2016).

### Software

The model was constructed using COPASI (Hoops et al., 2006) and EasyModel (Bartolomé et al., 2019). It was further analyzed using Mathematica (Wolfram Research Inc, 2021) to stabilize the system, conduct sensitivity analysis, and scan parameters. The code that generates the models and the figures can be downloaded from https://www.dropbox.com/s/9u77nj5mgp12e1w/Supplementary%20Data%20S1.rar?dl=0 as a Mathematica notebook.

## Results

### Data-driven model improvement

We built the alternative models using parameter values collected from various plants (Table 1). As such, and in order to describe the situation in maize more accurately, the parameter values needed adjustment. To do so, we gathered quantitative experimental data in maize for metabolites in the pathway and then tested to see the minimal changes in parameter values that would allow the model to generate those metabolite levels while maintaining a stable and robust steady state, which are hallmarks of good model quality.

We only found experimental measurements for BCAR in white maize lines (Zhu et al., 2008). Zhu et al. (2008) reported that the BCAR concentration in the South African elite white maize variety of M37W ranges from 10^–4^ (wild-type maize) to 10^–1^ (maize genetically transformed to produce carotenoids). The models using the basal parameter values can only produce half of the experimentally measured BCAR. Thus, to better approximate reality, the flux accounting for the production of BCAR (which is represented by k_0_) must be increased in the models. Yet, increasing k_0_ by an amount that produces the appropriate levels of BCAR leads to an unstable steady state (Figure 2). This is shown by the real parts of eigenvalues becoming positive after a certain threshold for a given parameter (Supplementary Figures 1, 2). For example, increasing the influx of BCAR above 10^–8^ will make the eigenvalue suddenly increase and approach a positive value hence resulting in an unstable system (Supplementary Figures 1A, 2A).

As such, we needed to determine which parameters could reasonably be adjusted to stabilize the steady state of the alternative models while maintaining BCAR levels that are consistent with experimental observation.

### Theory-driven model improvement

Stabilizing the steady state of the models and making the metabolite concentrations consistent with experimentally determined concentrations were done in two ways. First, we identified the parameters whose values could be changed to make the levels of BCAR consistent with experimental levels while stabilizing the steady state. Then, we investigated if there might be potential feedback regulatory loops that have yet to be experimentally characterized and could help in stabilizing the steady state.

#### Parameter adjustments

To stabilize the steady state of the alternative models we scanned the values for each parameter independently over six orders of magnitude, as described in methods and summarized in Figure 2. For each set of parameter values, we calculated the steady state of the model and the eigenvalues of that steady state to determine its stability (Supplementary Figures 1, 2). All eigenvalues should have negative real parts for stable steady states and thus represent physiological situations.

Supplementary Figure 1 shows the real part of the greatest eigenvalue changes as each parameter changes. This analysis revealed that changing the parameter values of k_0_, D27, K_*M D*27_, and CCD8 to those given in Table 1 provides the minimum intervention parameter set that stabilizes the steady state of Model AB while maintaining BCAR levels that are consistent with experimental determinations. The same goes for Model AC, where the parameter values in Table 2 are within the bounds of stability presented in Supplementary Figure 2.

#### Feedback regulation

Inhibitory feedback regulation is prevalent in biosynthetic pathways (Alves and Savageau, 2000). Still, we found no reported evidence for such regulation in SL biosynthesis. However, such a type of regulation is known to stabilize steady states (Alves and Savageau, 2000). As such, we investigated the possibility that, while yet unknown, such feedback could be present in the system. To do so, we performed the following *in silico* experiment.

**FIGURE 3:**
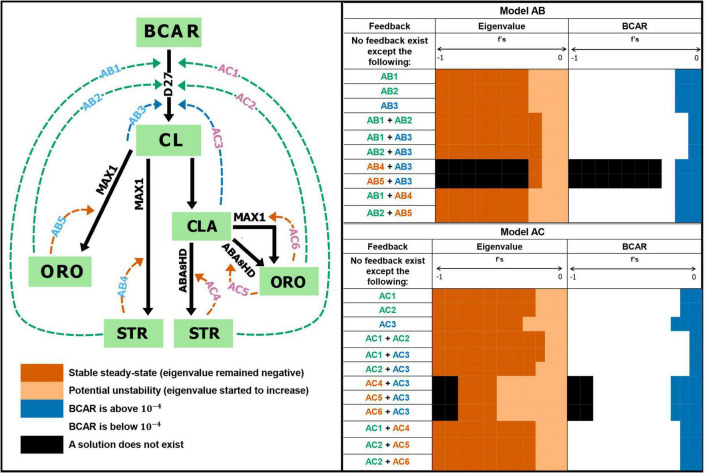
Effect of overall feedback regulation on strigolactone biosynthesis. Hypothetical inhibitory feedback was added to the models and the steady state was calculated. Feedback regulation is tested from –1 (strong negative feedback) to 0 (no feedback). Tested feedback combinations in Model AB: AB1, AB2, AB3, AB1 + AB4, AB2 + AB5, AB1 + AB2, AB1 + AB3, and AB2 + AB3. Tested feedback combinations in Model AC: AC3, AC1, AC2, AC2 + AC3, AC1 + AC4, AC2 + AC5, AC2 + AC6, AC1 + AC2, and AC1 + AC3. We find that maintaining experimental BCAR levels and stable steady state requires the absence of inhibitory feedback. See Supplementary Figures 3–6 for details.

First, we created the alternative models described in Figure 1. Then, for each of the models, we tested the effect of adding feedback loops, one at a time, on the steady-state stability and levels of BCAR, as portrayed in Figure 3. This was done by setting boundaries to the strength of the feedback effect on each reaction. We represent this feedback strength by the f parameters in Equations 4-6: *f* = −1 represents strong feedback, while *f* = 0 represents no feedback. Then, we scan this interval with jumps of 0.01 and calculate the steady state of the model for each set of parameter values. Finally, for each calculated steady state we check for stability and to determine whether BCAR levels are within the experimentally determined range of 10^–4^ to 10^–1^ (Zhu et al., 2008).

We test Models AB and AC for the dynamic effect that the possible existence of negative feedback (Alves and Savageau, 2000) to the first reaction from the end-products (ORO and STR) and branch point metabolites (CL or CLA) might have on the dynamics of the system. Figure 3 schematically illustrates all inhibitory feedback interactions we tested and qualitatively summarizes the effect of those feedback interactions on the dynamics of the models. Supplementary Figures 3, 4 show the impact of the various feedback interactions on steady state stability and BCAR concentration. By and large, if the feedback strength increases, the concentration of BCAR is either unaffected or decreases well below experimental levels. Similarly, weak feedback interactions either have no effect or decrease the stability of the steady state. As the strength of the feedback increases, that stability becomes similar to that of the basal models with no feedback.

We also analyzed the effect that the simultaneous existence of two feedback interactions at the same time would have in the dynamics of the models. Results are shown in Supplementary Figures 5, 6. Combining feedback interactions lead to qualitatively the same result as in applying only the individual feedback.

Finally, we note that we constrained the parameters of Models AB and AC to understand if evolution could achieve stable steady states and maintain BCAR levels in the presence of feedback. To do so we adjusted the rate constant of the reaction to which we added the feedback, in order to maintain the same steady state flux. Then, we recalculated the eigenvalues of this constrained system. We observed that the stability of the steady state decreases as the feedback strength increases, as was the case for the unconstrained simulations (Supplementary Figures 7, 8). Together, these results strongly suggest that inhibitory feedback regulation of the enzyme activity should not be present in the pathway.

### Model analysis

#### Local sensitivity analysis

**FIGURE 4:**
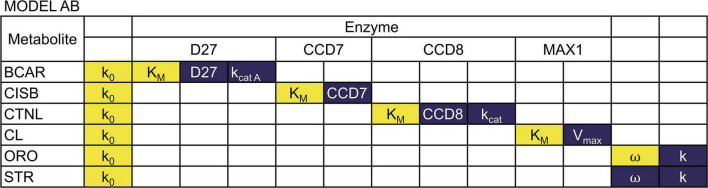
Sensitivity of metabolite concentrations to changes in parameter values for Model AB. Blue indicates that increasing the parameter by 1% will increase the metabolite concentration by more than 1%, while yellow indicates that increasing the parameter by 1% will decrease the metabolite concentration by at least 1%.

**FIGURE 5:**
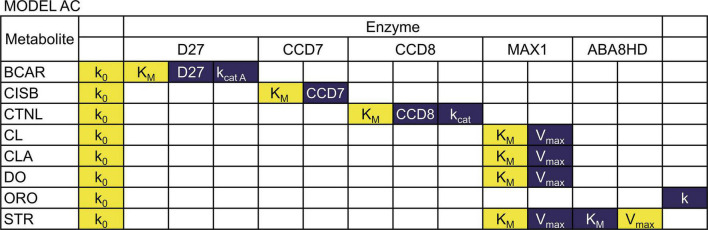
Sensitivity of metabolite concentrations to changes in parameter values for Model AC. Blue indicates that increasing the parameter by 1% will increase the metabolite concentration by 1%, while yellow indicates that increasing the parameter by 1% will decrease the metabolite concentration more than 1%.

We performed a sensitivity analysis of the two models as described in the methods. The results are summarized in Figures 4, 5 (see Supplementary Tables 1, 2 for full results).

We found that in both models, all concentrations are directly proportional to the rate constant of the BCAR production reaction. The substrate concentration of each reaction is, in general, inversely proportional to the values of k_*cat*_, V_*max*_, and enzyme concentration of that reaction. That concentration is also directly proportional to the K_*M*_ of the reaction. The sensitivity of the reaction products to the same parameters is negligible. We also find that the amount of each strigolactones is proportional to the branching parameter ω in Equation 5. If ω increases, ORO increases, and STR decreases in direct proportion to the change in ω.

Each concentration has small sensitivities to all other parameters in Model AB. In contrast, in Model AC, we find that the concentrations of CL, CLA, DO, STR strongly depend on parameters K_*MMAX*1_ and V_*maxMAX*1_. In addition, only the concentration of STR is sensitive to K_*MABA*8*HD*_ and V_*maxABA*8*HD*_. However, the effect of K_*MABA*8*HD*_ and V_*maxABA*8*HD*_ on STR is the opposite. The concentration of STR is inversely proportional to K_*MABA*8*HD*_ and directly proportional to V_*maxABA*8*HD*_.

**FIGURE 6:**
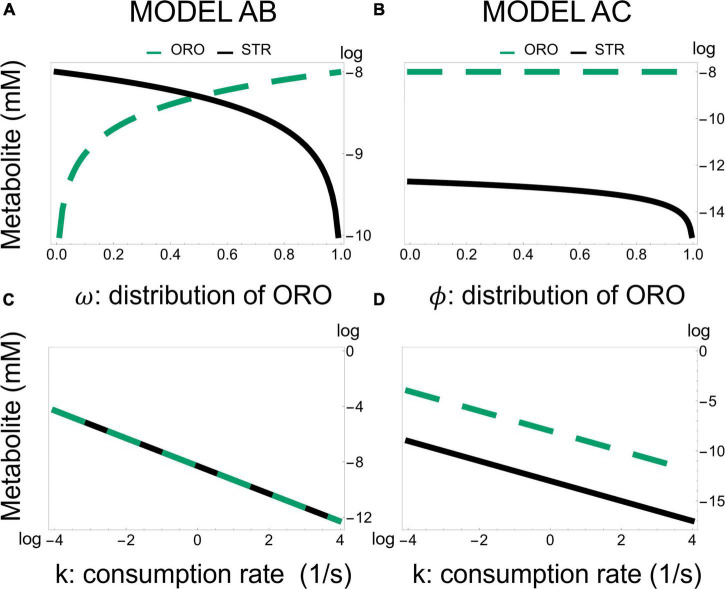
Effect of changing the fraction of flux going from BCAR to strigol-type or orobanchol-type of strigolactones in Models AB and AC. Model AB—Production of ORO and STR as a function of the flux branching parameter ω and diffusion of strigolactones, represented by k. Model AC—Production of ORO and STR as a function of the flux branching parameter ϕ and diffusion of strigolactones, represented by k. Panel **(A,B)** are semi-log plots (where y-axis is in log) while panel **(C,D)** are log-log plots.

To understand if the local sensitivity analysis we performed could be extrapolated to a global sensitivity analysis we scanned each parameter by at least four orders of magnitude about its normal value, calculating the new steady state values. For Model AB, in all cases, we found that metabolites changed as predicted from the local sensitivity analysis (Supplementary Table 1). We note that changing the value of the bifurcation parameter ω has the strongest effect on the balance of STR/ORO that is produced. As ω approaches 1 (0), only ORO (STR) is produced (Figure 6). For Model AC, we also found that the global sensitivity analysis is consistent with the local sensitivity analysis.

**FIGURE 7:**
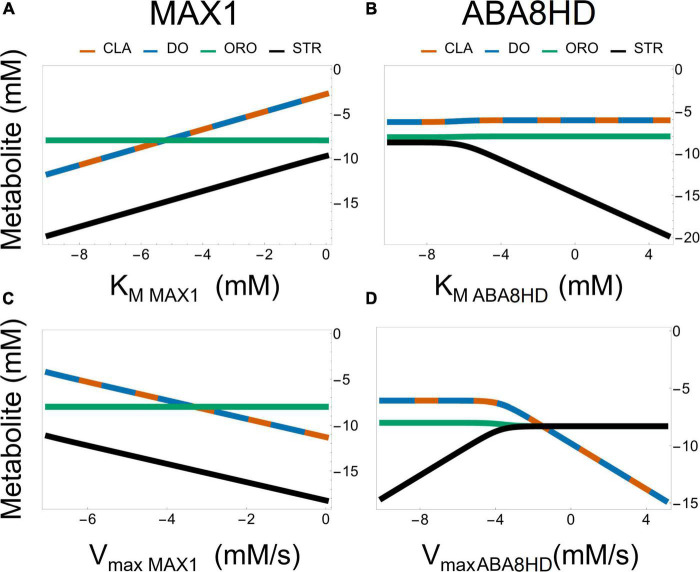
Effect of parameter values from the enzyme reactions driven by MAX1 [left panel **(A,C)**] and ABA [right panel **(B,D)**] on the metabolic concentrations of CLA, DO, ORO, and STR in Model AC. All plots are in log-log plane.

A striking difference between Models AB and AC is that the bifurcation parameter [ω in Equation 5 for model AB and ϕ in Equation 6 for model AC] has a weaker effect on the ratio STR/ORO being produced in Model AC (Figure 6). The concentration of ORO in Model AC is less sensitive to parameter changes than in Model AB, as it ranges only from 10^–8⋅3^ to 10^–8^ despite considerable changes in the K_*M*_’s and V_*max*_’s of both MAX1 and ABA8HD. This provides a differentiating feature between Models AB and AC. Moreover, Figure 7 shows the effect of all the parameters involved in MAX1 and ABA8HD on the metabolite concentrations of CLA, DO, ORO, and STR.

## Discussion

While mathematical modeling has been around for decades, it only recently became a cornerstone on which policymaking extensively relies due to the pandemic caused by the SARS-CoV-2 virus. This type of modeling provides a description of biological systems that can be accurately interrogated through simulation to reveal the differences in the dynamic behavior of alternative mechanisms, even before those differences can be measured directly (Schwiening, 2012). This can help in predicting how alternative designs for a network or pathway will affect the dynamic behavior of the system being modeled, which might help in reconstructing less well-known molecular pathways. However, despite several successful works, its application is still in the early stages in plants (Schwiening, 2012).

A complete characterization of the strigolactone biosynthesis pathway is still lacking. While the initial steps of the pathway are clear, the complete set of individual reactions in the SL production pathway(s) remains unclear. We combined available experimental information with mathematical modeling to investigate how possible alternative structures for the pathway would affect the dynamics of SL biosynthesis. Building the models required understanding the essential features of SL biosynthesis. The first part of the pathway is well-established (Alder et al., 2012). Still, there is limited knowledge regarding the individual reaction steps and enzymes that transform CL or CLA into SL and/or ORO. After extensive literature analysis, we identified two possible alternative pathways that are consistent with experimental information. These are represented by models AB and AC in Figure 1. Model AB is a more straightforward pathway, where the flux branching between ORO and STR is proposed to occur at the level of CL (as portrayed in Figure 1B). In contrast, Model AC was built based on evidence that CL is converted into CLA (Abe et al., 2014), ORO, and STR. In addition, Model AC includes an additional flux branch that produces only ORO (Yoneyama et al., 2018; Yoneyama and Brewer, 2021). Cytochrome P450 enzymes catalyze reactions in both pathways. Often, these enzymes have broad specificity in plants. We then used simulation to identify commonalities and differences in the dynamic behavior of the alternative models.

Both models predict that the most significant effect on the production of ORO and STR is achieved by modifying the influx of BCAR into the pathway (Supplementary Figures 7–11 and Supplementary Tables 1, 2), rather than changing the amount of enzyme in any intermediate step of the pathway. In addition, changing the ratio of ORO vs. STR produced in the pathway can be achieved by modulating the activity of enzymes after the branching points represented in Figure 1, as illustrated in Figures 6, 7.

Commonalities between the dynamic behavior stop here. The details about the best strategies for modulating differential production STR and ORO differ between models AB and AC. Model AB predicts concentration ranges for STR and ORO that can be similar to each other. In model AB it is possible to divert all flux toward the synthesis of either STR or ORO (Figure 6). In contrast, in model AC, ORO is produced at approximately the same rate, independently of the flux branching. In contrast, Model AC predicts that ORO concentration can only change over less than one order of magnitude and is orders of magnitude higher than that of STR, which can change over five orders of magnitude.

Another difference is that model AB points to changing the specificity of MAX1 as the primary determinant of the ratio between STR and ORO being produced (Figures 6, 7). Model AC suggests that changing the amount of ABA8HD enzyme is also essential to determining the balance between the concentrations of STR and ORO (Figures 6, 7). Thus, modulating the expression of MAX1 and ABA8HD in the plant and measuring the effects on the production of STR and ORO could elucidate which of the two models is closer to reality.

These differences can be used in future research to experimentally differentiate the alternative pathways. Once the correct pathway is identified, the model can then be used to rank potential genetic modifications that could change the ratio of STR/ORO and decrease the risk of inducing germination of local parasitic plants.

An interesting aspect of SL biosynthesis is that, while many biosynthesis pathways have negative feedback from their final product to the first enzyme of the pathway (overall feedback), this feedback seems to be absent in SL biosynthesis. When this was investigated, it became apparent that if biochemical regulation exists, it is superseded by circadian regulation (Pan et al., 2009; Khan et al., 2010). Still, feedback regulation of enzyme activity by intermediates or end products of a pathway provides many metabolic advantages (Alves and Savageau, 2000). As such, it seemed plausible that such feedback might exist and not have been observed yet. Thus, we tested what effect we could expect inhibitory feedback regulation to have in the dynamics of SL biosynthesis.

Surprisingly, our results strongly suggest that inhibitory feedback regulation does not exist in the SL biosynthetic pathway. The existence of that feedback regulation would create unstable steady states that would make it hard for the plants to develop properly. In addition, they would decrease the concentration of pathway substrate to levels well below those observed experimentally by Zhu et al. (2008). We observe the same type of behavior when combining possible alternative feedback inhibitory interactions. Thus, unlike amino acid biosynthesis, in which negative feedback regulation of the flux by the amino acid creates a pathway that is driven by demand, SL biosynthesis is driven by the supply of substrate to the pathway.

Carotenoids are the substrate of the SL biosynthesis pathway. They are also precursors of other developmental hormones, of photo-protection and photosynthetic pigments (Nisar et al., 2015). Thus, from an evolutionary point of view, it makes sense that the biosynthesis of SL hormones that regulate plant development are supply driven, because the pathway substrate availability is also linked to the plant’s ability to synthesize other molecules that are important in later stages of its development.

### Future perspective

Results obtained from the mathematical model implied that modulating strigolactone levels in cereals is possible by increasing the substrate levels of the pathway. Changing the ratio of STR/ORO produced could be achieved by modulating enzyme activities after the flux branching that leads to each strigolactone type. Our results strongly suggest that feedback regulation does not exist in the pathway, as it is not possible to reach experimental BCAR concentrations while simultaneously reaching a stable steady state. In addition, the next step in our study is to couple strigolactone biosynthesis to root development and build a combined model considering the pleiotropic effects of strigolactone to root growth and development.

## Data availability statement

The original contributions presented in this study are included in the article/Supplementary material, further inquiries can be directed to the corresponding author.

## Author contributions

AL and RA designed the experiments and wrote the first version of the manuscript. AL performed the experiments with the assistance of RA and OB. All authors analyzed the results, contributed to their discussion, and approved the final version.
