# Supplementary material for: A mathematical model for strigolactone biosynthesis in plants
Source: Front Plant Sci. 2022 Sep 2;13:979162. doi: 10.3389/fpls.2022.979162 (PMC9480829; doi:10.3389/fpls.2022.979162)
Supplement: Supplementary file 1 [file Table_1.docx]

**Supplementary Table 1.** Results of relative sensitivity analysis for Model AB

| Metabolite | k_0_ | K_M D27_ | D27 | k_cat D27A_ | K_M CCD7_ | CCD7 | K_M CCD8_ | CCD8 | k_cat CCD8_ | K_M MAX1_ | V_max MAX1_ | $\omega$ | k |
| --- | --- | --- | --- | --- | --- | --- | --- | --- | --- | --- | --- | --- | --- |
| BCAR | 1 | 1 | -1 | -1 |  |  |  |  |  |  |  |  |  |
| CISB | 1 |  |  |  | 1 | -1 |  |  |  |  |  |  |  |
| CTNL | 1.5 |  |  |  |  |  | 1 | -1.5 | -1.5 |  |  |  |  |
| CL | 1 |  |  |  |  |  |  |  |  | 1 | -1 |  |  |
| ORO | 1 |  |  |  |  |  |  |  |  |  |  | 1 | -1 |
| STR | 1 |  |  |  |  |  |  |  |  |  |  | -1 | -1 |
