# Supplementary material for: A mathematical model for strigolactone biosynthesis in plants
Source: Front Plant Sci. 2022 Sep 2;13:979162. doi: 10.3389/fpls.2022.979162 (PMC9480829; doi:10.3389/fpls.2022.979162)
Supplement: Supplementary file 2 [file Table_2.docx]

**Supplementary Table 2.** Results of relative sensitivity analysis for Model AC

| Metabolite | k_0_ | K_M D27_ | D27 | k_cat D27A_ | K_M CCD7_ | CCD7 | K_M CCD8_ | CCD8 | k_cat CCD8_ | K_M MAX1_ | V_max MAX1_ | K_M ABA8HD_ | V_max ABA8HD_ | k |
| --- | --- | --- | --- | --- | --- | --- | --- | --- | --- | --- | --- | --- | --- | --- |
| BCAR | 1 | 1 | -1 | -1 |  |  |  |  |  |  |  |  |  |  |
| CISB | 1 |  |  |  | 1 | -1 |  |  |  |  |  |  |  |  |
| CTNL | 1.5 |  |  |  |  |  | 1 | -1.5 | -1.5 |  |  |  |  |  |
| CL | 1 |  |  |  |  |  |  |  |  | 1 | -1 |  |  |  |
| CLA | 1 |  |  |  |  |  |  |  |  | 1 | -1 |  |  |  |
| DO | 1 |  |  |  |  |  |  |  |  | 1 | -1 |  |  |  |
| ORO | 1 |  |  |  |  |  |  |  |  |  |  |  |  | -1 |
| STR | 1 |  |  |  |  |  |  |  |  | 1 | -1 | -1 | 1 |  |
